# Multicentric Reticulohistiocytosis Presenting with Papulonodular Skin Lesions and Arthritis Mutilans

**DOI:** 10.1155/2013/201563

**Published:** 2013-03-10

**Authors:** Raya Saba, Shawn G. Kwatra, Bishwas Upadhyay, Aibek E. Mirrakhimov, Farah N. Khan

**Affiliations:** Department of Internal Medicine, Saint Joseph Hospital, 2900 N. Lake Shore, Chicago, IL 60657, USA

## Abstract

Multicentric reticulohistiocytosis is a rare multisystem disorder of unknown etiology that is characterized by erosive polyarthritis and papulonodular lesions on the skin, mucous membranes, and internal organs. We report the case of a 54-year-old female who was misdiagnosed as having rheumatoid arthritis and underwent numerous joint replacement surgeries for progressively destructive arthritis in her hands, shoulders, hips, and knees. The patient finally received a diagnosis of multicentric reticulohistiocytosis after histopathological examination of the patient's left knee arthroplasty which revealed a diffuse histiocytic infiltrate, multinucleated giant cells, and finely granulated eosinophilic cytoplasm with a ground-glass appearance.

## 1. Introduction 

Multicentric reticulohistiocytosis (MRH) is a rare multisystem disorder of unknown etiology, characterized by erosive polyarthritis and papulonodular lesions on the skin, mucous membranes, and internal organs. MRH is the most destructive chronic inflammatory arthritis, manifesting more aggressively than either rheumatoid arthritis (RA) or psoriatic arthritis [1], and progressing to arthritis mutilans in 45% of cases [2]. The onset of the disease is usually in the forth decade of life, affecting females two to three times more often than males [3, 4], and presenting insidiously with joint symptoms preceding skin manifestations in the majority of patients. Herein, we describe the case of a patient found to have multicentric reticulohistiocytosis who was previously misdiagnosed as having rheumatoid arthritis for several years. 

## 2. Description of Case

A 54-year-old African-American female with a history of multiple joint replacements presented to clinic complaining of severe diffuse arthritis. She reported progressive morning stiffness and arthralgias in her knees that first started 10 years prior, followed shortly by the development of skin nodules on the dorsum of both hands. Since this time, multiple other joints became affected including her bilateral hands, hips, and shoulders. 

She was initially seen by a rheumatologist 10 years ago and diagnosed with RA. Over the course of her disease, she tried numerous therapies including ibuprofen, prednisone, etanercept, methotrexate, and adalimumab. However, she had a poor response to these medications, and her arthritis continued to worsen. She eventually underwent bilateral hip and knee replacement surgeries to improve her deteriorating functional ability. 

**Figure 1:**
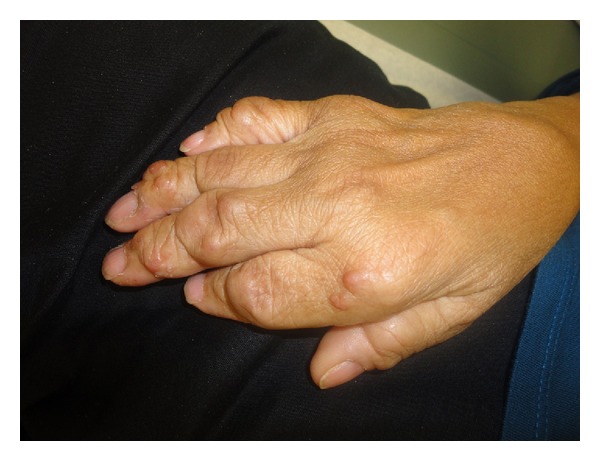
Reddish-brown papulonodular lesions on the dorsum of the hand.

On physical exam, the patient had multiple nonpruritic reddish-brown papulonodular lesions on the dorsum of the hands that were present for the past 8 years (Figure 1). The skin rash started approximately 2 years after the onset of symptoms. She also had eyelid xanthelasmas along with arthritic deformities involving the metacarpophalangeal (MCP), proximal interphalangeal (PIP), and distal interphalangeal (DIP) joints. Other features observed included telescoping of the digits bilaterally, ulnar deviation of the wrists, and limited abduction and flexion of the shoulders.

Extensive laboratory workup was significant for a microcytic hypochromic anemia, elevated erythrocyte sedimentation rate (ESR) and C-reactive protein (CRP), and an abnormal lipid panel. The following tests were within normal limits: antinuclear antibodies, rheumatoid factor, anticyclic citrullinated protein antibody, liver function tests, and thyroid studies. 

**Figure 2:**
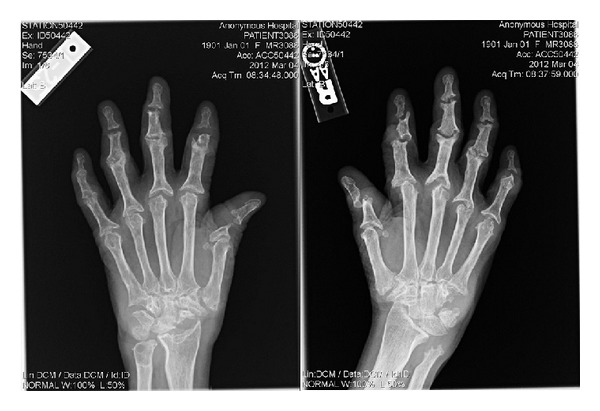
X-ray of the hand showing the “pencil-in-cup” phenomenon and narrowing of the joint spaces.

**Figure 3:**
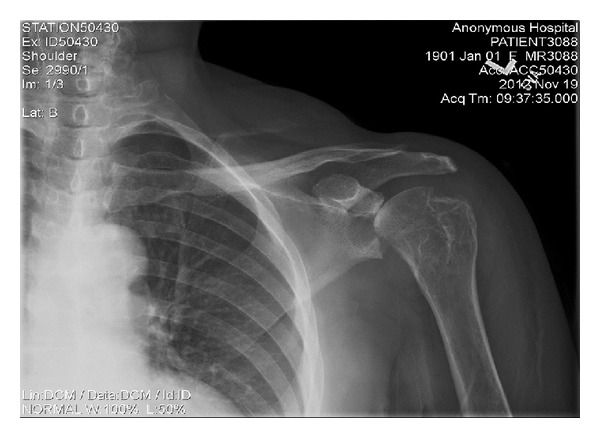
X-ray of the shoulder displaying multiple erosions of the acetabulum, humeral head, and acromioclavicular articulation.

X-ray of the hand showed severe diffuse destruction of the carpometacarpal, metacarpophalangeal, and interphalangeal joints with widening of joint spaces and expanded bases of proximal and middle phalanges giving the appearance of a pencil-in-cup deformity (Figure 2). Shortening and complete resorption of certain phalanges was also seen along with interphalangeal joint fusion and subluxation. There was also significant periarticular osteoporosis, narrowing of the joint spaces, and new bone formation. X-ray of the shoulder revealed destructive changes and multiple erosions of the acetabulum, humeral head, and acromioclavicular articulation, without any associated fracture or dislocation (Figure 3). These findings were consistent with arthritis mutilans.

**Figure 4:**
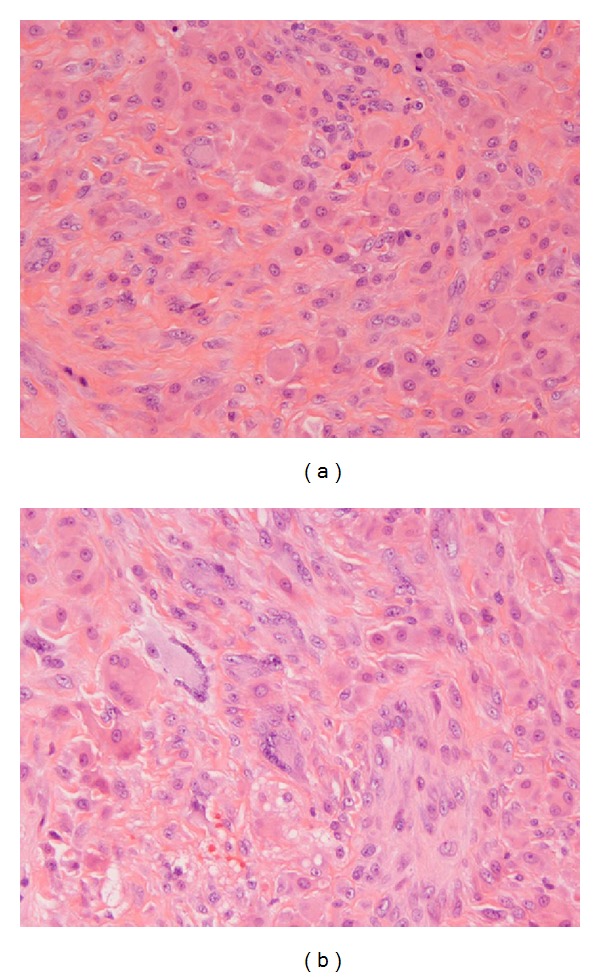
Histiocytic infiltration with multinucleated giant cells.

**Figure 5:**
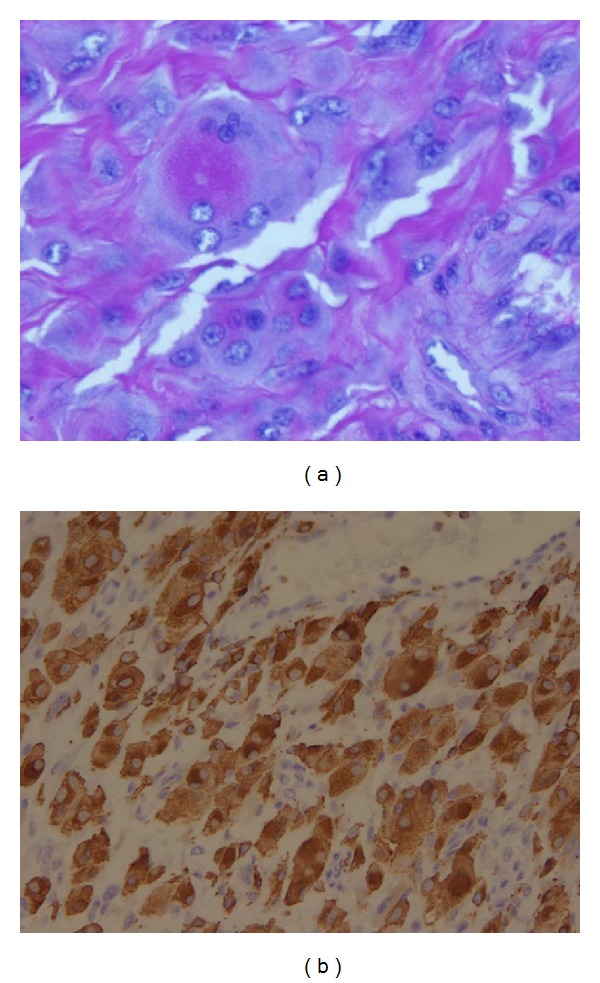
(a) Histiocytes with PAS material; (b) histiocytes staining positive for CD68.

Finally, histopathological examination of the patient's left knee arthroplasty reported marked histiocytic infiltration with multinucleated giant cells (Figure 4). Other features noted include a finely granulated eosinophilic cytoplasm with a ground-glass appearance, vesicular nuclei, hyperplastic synovial tissue, and chronic inflammation with fibrin deposition. Histiocytes and histiocytic giant cells contained periodic acid Schiff (PAS) reactive material (Figure 5(a)) and stained positive for CD68 (Figure 5(b)).

The patient's combination of clinical presentation, laboratory testing, imaging, and histopathology was consistent with a diagnosis of multicentric reticulohistiocytosis. Due to poor compliance, the patient failed to undergo screening for an associated malignancy.

Treatment regimens have been adjusted frequently. She failed a combination of adalimumab and methotrexate as well as a trial of azathioprine. Her current regimen consists of etanercept, methotrexate, and prednisone, which provides symptomatic relief including decreased stiffness, edema, and pain. This treatment regimen has unfortunately failed to resolve her irreversible arthritic deformities.

## 3. Discussion

MRH is a rarely diagnosed rheumatologic condition. While only approximately 250 cases have been reported worldwide, this is likely to be a significant underrepresentation of the disease. Owing to its rarity, the diagnosis can be challenging, and many patients are misdiagnosed with more common causes of erosive polyarthritis, such as RA. Hence, increased awareness plays a crucial role, and a high index of suspicion is warranted by clinicians in patients presenting with erosive polyarthritis. Helpful clues (which were all present in our case) include the development of characteristic skin lesions, X-ray findings including involvement of the distal interphalangeal joints (DIP), rapid progression to arthritis mutilans, and confirmatory histopathology findings.

Arthritis associated with MRH is inflammatory in nature and tends to be symmetrical. There is maximal involvement of the interphalangeal joints of the hands that can lead to the disabling “accordion” or “opera glass” hand [3]. Involvement of the distal interphalangeal (DIP) joints is present in 75% of patients, representing an important diagnostic clue in distinguishing MRH from other types of erosive arthritis, mainly rheumatoid arthritis [5]. 

Skin involvement is a characteristic of MRH and involves multiple flesh-colored to reddish-brown nonpruritic firm papules and nodules, ranging in size from a few millimeters to 2 cm in size. Most of these lesions are commonly located over the dorsal aspects of the hands, the elbows, and on the head. Systemic complaints have also been observed and most often affect the pulmonary, cardiac, and muscular systems.

MRH has been described in association with various conditions: hyperlipidemia (30–58%), a positive skin tuberculin test (12–50%), and many autoimmune diseases (5–20%) including RA, Sjogren's syndrome [6], primary biliary cirrhosis [7], and systemic vasculitis [8]. A higher association with underlying internal malignancy has been reported in up to 25% of MRH patients with hematological, breast, and stomach carcinomas being the most common [9, 10]. It has, thus, been classified by several authors as a paraneoplastic syndrome, occurring in close proximity to the onset of a new neoplasm or the recurrence of a previously diagnosed one, and regressing upon treatment of the primary tumor [11]. A thorough investigation to exclude malignancy is highly recommended in patients with newly diagnosed MRH.

Laboratory investigations lack specificity and diagnostic relevance. These investigations may reveal a microcytic anemia, elevated ESR, elevated CRP, and hyperlipidemia. Imaging, on the other hand, plays a key diagnostic role. Radiologic findings include well-circumscribed marginal erosions, widening of joint spaces, loss of cartilage, and resorption of subchondral bone. In contrast to other types of inflammatory arthritis, periarticular osteopenia and heterotrophic new bone formation are rarely reported [1, 12].

The diagnosis is confirmed histologically, with a skin or synovial tissue biopsy demonstrating pleomorphic mononuclear histiocytes, multinucleated giant cells, and eosinophilic ground-glass cytoplasm that is PAS positive [3]. 

These cells have also been shown to stain positively with the osteoclast markers tartrate-resistant acid phosphatase (TRAP) and Cathepsin K. This finding, along with good response to treatment with bisphosphonates, provides support for the notion of MRH being a systemic osteoclastic disease [13].

Although symptoms associated with MRH may spontaneously remit within 5–10 years of diagnosis, aggressive treatment is recommended to avoid irreversible sequelae of the active inflammation period. Due to the limited number of cases, treatment continues to be experimental. Remission has been achieved in patients with MRH with methotrexate, chlorambucil, and cyclophosphamide. These drugs have been used in various combinations with adjuvant nonsteroidal anti-inflammatory agents, hydroxychloroquine, and corticosteroids [14]. MRH has also shown a successful response to azathioprine [15] and leflunomide [16]. More recent data show excellent results achieved with antitumor necrosis factor (TNF*α*) therapy including etanercept [17], infliximab [18], and adalimumab [19]. Finally, improvement has also been noted with bisphosphonates, including alendronate [20] and zoledronic acid [13]. 

## 4. Conclusion

Clinicians should have a high index of suspicion for MRH in patients presenting with erosive polyarthritis and papulonodular skin lesions. Helpful clues for diagnosis include involvement of the DIP, rapid progression to arthritis mutilans, and characteristic histopathological findings including a diffuse histiocytic infiltrate, multinucleated giant cells, and finely granulated eosinophilic cytoplasm with a ground-glass appearance.
